# Qualitätssicherung in medizinischen Laboratorien – Eine Unentbehrlichkeit mit Nutzen und Risiken

**DOI:** 10.1007/s00103-022-03502-5

**Published:** 2022-02-15

**Authors:** Janine Kleymann-Hilmes, Sophia Brünschwitz, Michael Müller

**Affiliations:** 1 ZV 6.2 Qualitätsmanagement, Robert Koch-Institut, Seestraße 10, 13353 Berlin, Deutschland; 2 Akkreditierte Labore in der Medizin – ALM e. V., Berlin, Deutschland

**Keywords:** SARS-CoV-2-Pandemie, Qualitätssicherung, Qualitätsmanagement, Medizinische Labore, Medizinprodukteüberwachung, SARS-CoV‑2 pandemic, Quality assurance, Quality management, Medical laboratories, Medical device monitoring

## Abstract

Der Stellenwert von Qualitätssicherung (QS) und Qualitätsmanagement (QM) in medizinischen Laboratorien ist gerade seit Beginn der SARS-CoV-2-Pandemie ein Thema in vielen politischen und öffentlichen Diskussionen. Einheitliche Standards werden benötigt, um Vergleichbarkeit nicht nur in Deutschland unter den medizinischen Laboratorien zu schaffen, sondern auch über die Landesgrenze hinaus. Dafür gibt es in Deutschland zwei federführende Systeme: die Richtlinie der Bundesärztekammer zur Qualitätssicherung laboratoriumsmedizinischer Untersuchungen (Rili-BÄK) und die Akkreditierung nach der Norm DIN EN ISO 15189.

Der Artikel dient der Sensibilisierung für Qualität in medizinischen Laboren zur Gewährleistung der Patientensicherheit durch eine kompetente Diagnostik am Patienten. Es wurde eine Literaturrecherche u. a. von Gesetzestexten, Normen sowie weiterführenden Dokumenten und Veröffentlichungen des QM durchgeführt sowie Erfahrungswerte von Vertretern unterschiedlicher Institutionen berücksichtigt.

Ein umfassendes QM-System unterstützt die Arbeit in der medizinischen Laboratoriumsdiagnostik maßgeblich. Die Wahrung des Vertrauens in die Leistungsfähigkeit von medizinischen Laboratorien und die jeweils tragenden Einrichtungen ist für eine bestmögliche Versorgung der Bevölkerung von großer Bedeutung. Auch der Mehraufwand, der mit einer Akkreditierung einhergeht, ist mit Blick auf die Patientensicherheit gerechtfertigt, sollte jedoch keine zusätzliche Bürokratie erfahren.

## Abstract (en)

The importance of quality assurance (QA) and quality management (QM) in medical laboratories has been a topic in many political and public discussions, especially since the beginning of the SARS-CoV‑2 pandemic. Uniform standards are required in order to create comparability not only among medical laboratories in Germany, but also across national borders. There are two leading systems in Germany: The Guideline of the German Medical Association (Rili-BÄK) and accreditation according to DIN EN ISO 15189.

This article is intended to raise awareness of quality in medical laboratories to ensure patient safety through competent diagnostics on the patient. A literature search was carried out, including legal texts, standards, and further QM documents and publications. Furthermore, empirical values from representatives of various institutions are considered.

A comprehensive QM system significantly supports work in medical laboratory diagnostics. Maintaining trust in the efficiency of medical laboratories and the facilities that support them is of great importance for the best possible care for the population. The additional effort associated with accreditation is justified with regard to patient safety but should not experience any additional bureaucracy.

## Einleitung

Mit der SARS-CoV-2-Pandemie ist das Bewusstsein für den Stellenwert von Qualitätssicherung (QS) und Qualitätsmanagement (QM) in medizinischen Laboratorien wieder stärker geworden. Das Robert Koch-Institut (RKI) und auch die medizinischen Laboratorien, die als medizinische Laboratorien nach der Norm DIN EN ISO 15189 [1] und als Prüflaboratorien zum Teil nach der Norm DIN EN ISO/IEC 17025 [2] akkreditiert sein können, erreichte eine Vielzahl von Anfragen von Stakeholdern innerhalb Deutschlands und international, inwieweit ihre Diagnostik und Empfehlungen qualitätsgesichert seien.

Schon früh wurde in Deutschland ein international sehr beachtetes wie stark frequentiertes System der externen Qualitätssicherung zur SARS-CoV-2-PCR von der Gesellschaft zur Förderung der Qualitätssicherung in medizinischen Laboratorien e. V. (INSTAND e. V.) etabliert. Die Gewährleistung von korrekten Interpretationen fehlerfreier Testergebnisse wurde zu einem Thema in öffentlicher und politischer Diskussion. In Deutschland ist die Einrichtung eines Qualitätssicherungssystems nach dem Stand der medizinischen Wissenschaft und Technik für laboratoriumsmedizinische Untersuchungen gemäß § 9 der Medizinprodukte-Betreiberverordnung (MPBetreibV) verpflichtend [3]. In der Verordnung wird auf die Richtlinie der Bundesärztekammer zur Qualitätssicherung laboratoriumsmedizinischer Untersuchungen (Rili-BÄK) verwiesen, deren Einhaltung als ordnungsgemäße Qualitätssicherung verstanden wird. Darüber hinaus besteht die Möglichkeit der Akkreditierungen nach internationalen Normen. Dies ist in manchen Ländern verpflichtend, in anderen, wie z. B. in Deutschland, wiederum nicht oder nur in bestimmten Wirtschaftsbereichen.

Dieser Beitrag befasst sich mit der Qualitätssicherung von medizinischen Laboratorien in Deutschland und deren Standards durch die in Deutschland angewendeten Qualitätsmanagement- und Qualitätssicherungssysteme. Es wird u. a. auf das Aufwand-Nutzen-Verhältnis von QS-/QM-Maßnahmen und auf zukünftige Herausforderungen für die Weiterentwicklung von QS und QM eingegangen.

## Standards für die Qualitätssicherung medizinischer Laboratorien

Die verbindliche Grundlage für die QS in medizinischen Laboratorien ist in § 9 der MPBetreibV niedergelegt. Hier lautet es: „Wer laboratoriumsmedizinische Untersuchungen durchführt, hat vor Aufnahme dieser Tätigkeit ein Qualitätssicherungssystem nach dem Stand der medizinischen Wissenschaft und Technik zur Aufrechterhaltung der erforderlichen Qualität, Sicherheit und Leistung bei der Anwendung von In-vitro-Diagnostika sowie zur Sicherstellung der Zuverlässigkeit der damit erzielten Ergebnisse einzurichten. Eine ordnungsgemäße Qualitätssicherung nach Satz 1 wird vermutet, wenn Teil A der Richtlinie der Bundesärztekammer zur Qualitätssicherung laboratoriumsmedizinischer Untersuchungen beachtet wird“ [3]. Die genannte Richtlinie legt grundsätzliche Anforderungen an das QM und die QS laboratoriumsmedizinischer Untersuchungen fest. Die Einhaltung dieser Vorgaben bzw. der Nachweis, dass davon abweichende Regelungen im Labor als gleichwertig qualitätsgesichert gelten können, gilt für alle, die laboratoriumsmedizinische Untersuchungen durchführen, so auch für die medizinischen Laboratorien. Eine Überwachung von Einrichtungen, Arztpraxen und medizinischen Laboratorien erfolgt durch die je nach Bundesland zuständige Behörde. Dies ist z. B. in Berlin, Brandenburg [4] und Baden-Württemberg [5] das zuständige Landesamt für Mess- und Eichwesen, in Hessen das Fachzentrum für Produktsicherheit und Gefahrenstoffe des Regierungspräsidiums Kassel [6] und in Nordrhein-Westfalen die Bezirksregierung [7].

In Hinblick auf die labordiagnostische Leistungserbringung in der Patientenversorgung gelten für die medizinischen Labore weitere gesetzliche Grundlagen, die ebenfalls zu beachten und zudem auch von Bedeutung für den Erhalt der Vergütung für die erbrachten fachärztlichen Leistungen sind. Das Fünfte Buch Sozialgesetzbuch (SGB V; [8]) legt den Rahmen fest für:

- die Qualität und Wirksamkeit von Leistungen (§ 2),
- die gebotene fachliche Qualität von Leistungen (§ 70),
- die gesetzlichen Vorgaben zur Etablierung eines Qualitätssicherungssystems (§ 135a) sowie
- die Teilnahme an einrichtungsübergreifenden Maßnahmen zur Qualitätssicherung und zur Verbesserung der Ergebnisqualität (§ 137).

Es gibt eine Vielzahl weiterer Gesetze und Verordnungen, die sich am SGB V orientieren und enge Berührungspunkte zu Qualitätssicherung und Qualitätsmanagement labordiagnostischer Leistungen aufweisen, beispielsweise das Gesetz über technische Assistenten in der Medizin (MTA-Gesetz, § 9; [9]), das Arzneimittelgesetz (§ 20b, s. auch 8. Abschnitt „Sicherung und Kontrolle der Qualität“; [10]), die Medizinproduktebetreiberverordnung (§ 9; [3]), die Ärztliche Berufsordnung (§ 5; [11]), die Qualitätssicherungsrichtline des Gemeinsamen Bundesausschusses [12], die Richtlinie der Bundesärztekammer zur Qualitätssicherung laboratoriumsmedizinischer Untersuchungen (siehe u. a. Teil A Kapitel 7, 8; [13]), die Vereinbarung von GKV-Spitzenverband und Kassenärztlicher Bundesvereinigung zur Qualitätssicherung nach § 135 Abs. 2 SGB V zur Erbringung von speziellen Untersuchungen der Laboratoriumsmedizin sowie die Technischen Regeln für Biologische Arbeitsstoffe (TRBA) 100 (s. Kap. 4; [14]) und TRBA 250 [15].

Es lässt sich feststellen, dass in den fachärztlichen Gebieten der medizinischen Labordiagnostik, zu denen neben der Laboratoriumsmedizin die Humangenetik, die Mikrobiologie, die Virologie und die Infektionsepidemiologie sowie die Transfusionsmedizin gehören, Systeme zur Qualitätssicherung verbindlich etabliert sind. Der Nachweis einer in Deutschland freiwilligen Akkreditierung (Infobox 1) nach der speziell für medizinische Laboratorien geltenden internationalen Norm DIN EN ISO 15189 gilt zudem als Beleg dafür, umfassend und erfolgreich bestehende Vorgaben zur Qualitätssicherung einzuhalten. Die mit einer Akkreditierung, z. B. nach DIN EN ISO 15189, verbundenen kontinuierlich und umfassend zu absolvierenden Überwachungsphasen durch die Deutsche Akkreditierungsstelle (DAkkS) sind ein wichtiger Unterschied zur eher risikoorientiert ausgerichteten behördlichen Überprüfung der Einhaltung der Rili-BÄK, welche über die Eingliederung in die MPBetreibV als Richtlinie für eine ordnungsgemäße Qualitätssicherung gilt. Zwar ist die Vergütung von laboratoriumsmedizinischen Leistungen in der Versorgung gesetzlich Versicherter an den Nachweis der erfolgreichen Teilnahme an Ringversuchen (Eignungsprüfungen) im Rahmen der Abrechnung gebunden, jedoch stellt dieser Nachweis nur einen Teil wirksamer Maßnahmen zur Qualitätssicherung dar.

Zur Sicherung der Qualität können die weitaus höheren Standards der internationalen Normen verwendet werden. Das Qualitätsmanagement z. B. des RKI basiert auf folgenden 3 Normen:

- DIN EN ISO/IEC 17025 („Allgemeine Anforderungen an die Kompetenz von Prüf- und Kalibrierlaboratorien“),
- DIN EN ISO 15189 („Medizinische Laboratorien – Anforderungen an die Qualität und Kompetenz“),
- DIN EN ISO/IEC 17043 („Konformitätsbewertung – Allgemeine Anforderungen an Eignungsprüfungen“).

Eine Anwendung der DIN EN ISO/IEC 17025 oder der DIN EN ISO 15189 erfüllt zugleich die Grundsätze der ISO 9001 („Qualitätsmanagementsysteme – Anforderungen“). Die kompetente Umsetzung dieser Normen in Laboren wird durch eine Akkreditierung bestätigt, denn eine akkreditierte Diagnostik schafft Vertrauen bei den Adressaten der Berichte. Akkreditiert werden z. B. am RKI schwerpunktmäßig die Konsiliarlaboratorien (KL) und nationalen Referenzzentren (NRZ) nach DIN EN ISO 15189 und DIN EN ISO/IEC 17025. In Deutschland ist es für medizinische Laboratorien nicht gesetzlich gefordert sich nach DIN EN ISO 15189 akkreditieren zu lassen, ganz anders als z. B. in Frankreich. Hier ist es seit 2009 für alle medizinischen Labore verpflichtend. Auch im Vereinigten Königreich wurde mit Blick auf die Qualitätssicherung der medizinischen Diagnostik im Rahmen der SARS-CoV-2-Diagnostik während der Pandemie eine „Akkreditierungspflicht“ für SARS-CoV-2-Diagnostik in medizinischen Laboren eingeführt [16].

## Transparenter Umgang mit Informationen zur Qualität von Leistungserbringern

Im stationären Versorgungsbereich erstellen die Einrichtungen einen Qualitätsbericht, der über die Homepage auch der Öffentlichkeit zur Verfügung gestellt wird. Im ambulanten Versorgungsbereich wird seitens der Kassenärztlichen Bundesvereinigung ein zusammenfassender jährlicher Qualitätsbericht [17] veröffentlicht. Die medizinischen Labore selbst informieren in ihren Internetangeboten über Inhalt, Struktur und Prozess der eigenen Qualitätsmanagement- und Qualitätssicherungssysteme. Ob und in welchem Umfang das Informationsangebot von Patienten, Auftraggebern bzw. Betroffenen genutzt wird, kann nur im Rahmen von Erfahrungen grob geschätzt werden. Für Ausschreibungen, Beauftragungen und auch Überweisungen von Leistungen werden diese Informationen genutzt. Die Verständlichkeit der Inhalte ist für Patienten eher eingeschränkt gegeben. Ob hier eine laienverständlichere Erläuterung vorteilhaft sein kann, wäre im Rahmen von Befragungen und Studien zu untersuchen.

Akkreditierten Laboratorien ist es gestattet, das Akkreditierungssymbol zusammen mit der eigenen Akkreditierungsnummer auf den Befundberichten und im sonstigen Schriftverkehr zu verwenden, um damit die Nutzer über die erfolgreiche Absolvierung des Akkreditierungsprozesses zu informieren [18]. In der Regel wird über die Homepage der medizinischen Labore auch die Akkreditierungsurkunde inklusive Anlage mit Einzelauflistung der jeweilig akkreditierten Untersuchungen zur Information zur Verfügung gestellt.

**Figure 1:**
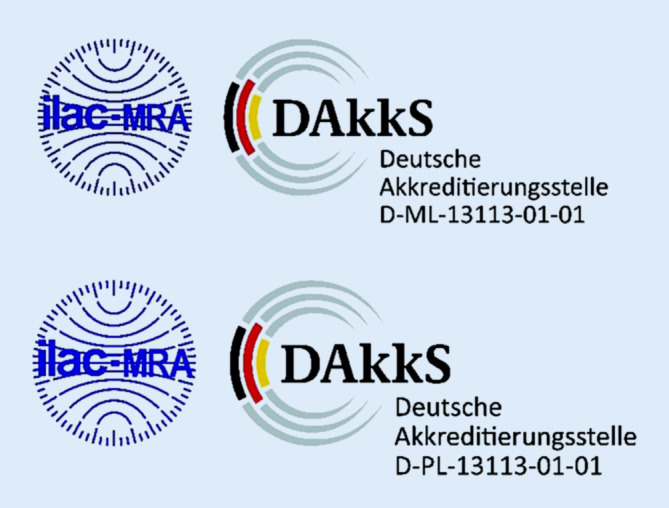

Mit den gegenseitigen internationalen Anerkennungsvereinbarungen „International Laboratory Accreditation Cooperation (ILAC) Mutual Recognition Arrangement (MRA)“ haben sich akkreditierte Laboratorien die nationale und internationale Anerkennung ihrer kompetenten medizinischen Leistungen in allen ILAC-Mitgliedsstaaten gegenüber den jeweiligen Stakeholdern gesichert. Über 70 Länder und regionale Organisationen sind in der ILAC als Internationale Vereinigung von Akkreditierungsstellen im Bereich der Laborakkreditierungen zusammengefasst. In Abb. 1 ist exemplarisch das DAkkS/ILAC-Symbol des RKI abgebildet. Dieses kombinierte Symbol kann auf Ergebnisberichten im Geltungsbereich der Akkreditierung abgebildet werden. Für den Empfänger der Berichte wird dadurch ersichtlich, welche der angewandten Methoden in den Geltungsbereich der Akkreditierung fallen. Es ist der Nachweis einer kompetenten und vertrauensvollen Labordiagnostik und dokumentiert die nationale und internationale Anerkennung in allen ILAC-Mitgliedsstaaten auf Grundlage der extern überprüften kompetenten medizinischen Leistungen. Es dient weltweit als Erkennungszeichen und wird zur Visualisierung der einheitlichen Standards verwendet.

Die Akkreditierungsurkunden samt Anhängen sind für die Öffentlichkeit in der Regel sowohl auf der Homepage des akkreditierten Laboratoriums als auch auf der Homepage der DAkkS einsehbar. Labore, welche die Dienstleistungen des Laboratoriums in Anspruch nehmen, haben über die Akkreditierungsurkunden einen Nachweis, dass die Ergebnisse, mit welchen sie weiterarbeiten, aus qualitätsgesicherter Diagnostik stammen.

## Verbesserungsprozesse durch Qualitätssicherung und Qualitätsmanagement

Aus Sicht der medizinischen Labore ist der kontinuierliche Verbesserungsprozess ein wichtiger Aspekt der seit den 1990er-Jahren in Deutschland zunehmend verbreiteten Akkreditierung medizinischer Labore. Er führte in allen Bereichen des Labors zu einer verstärkten Sensibilisierung gegenüber Faktoren, die die Qualität der Laboratoriumsuntersuchungen beeinflussen. Wichtige Bestandteile etablierter QM-Systeme, die Verbesserungsprozesse in Gang halten, sind das Reklamations- und Beschwerdemanagement, die Instrumente der Fehlerkorrektur inkl. Vorbeugemaßnahmen sowie das interne Auditwesen. Eine besondere Herausforderung stellt die „Messung des Effektes auf die Qualität der Patientenversorgung“ dar. Hierzu bedarf es der Entwicklung von tauglichen Qualitätsindikatoren und -messgrößen.

**Figure 2:**
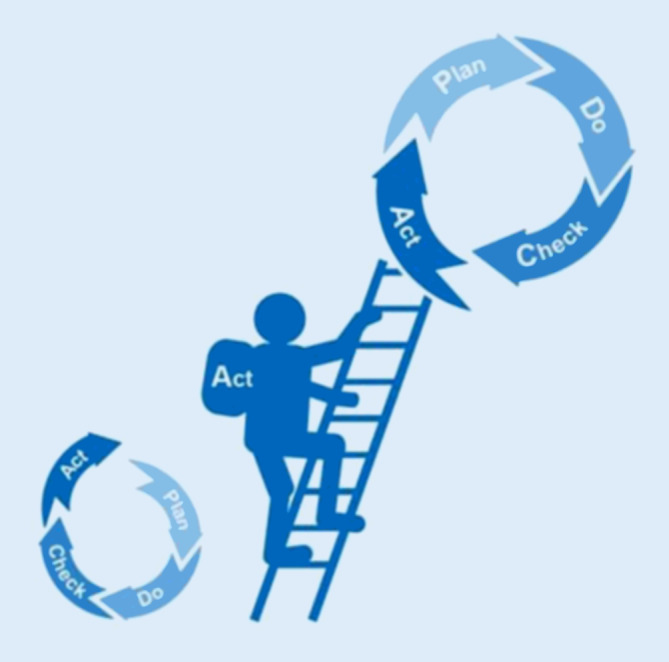

Über die Einführung gesetzlicher Vorgaben im SGB V sind Maßnahmen zur Qualitätssicherung sowie die Teilnahme an einrichtungsübergreifenden Maßnahmen Pflicht für medizinische Einrichtungen. Dabei gehören die medizinischen Labore mit zu den Vorreitern in Hinblick auf QS- und QM-Konzepte, die differenziert und auf eine bestmögliche Leistungserbringung ausgerichtet sind. Allgemein gilt: Wer ein QM-System nach der Rili-BÄK, ISO 9001, ISO 15189 oder ISO/IEC 17025 betreibt, der verpflichtet sich auch einem kontinuierlichen Verbesserungsprozess. Die Theorie dahinter kann durch den sog. Deming-Kreis, auch Plan-Do-Check-Act-(PDCA-)Zyklus genannt, visualisiert werden (Abb. 2). Dieser Zyklus aus „Planen“, „Durchführen“, „Überprüfen“ und „Handeln“ ermöglicht eine kontinuierliche Qualitätssteigerung der Labore. Neue Prozesse oder Prozessveränderungen werden vorab gründlich geplant, dann umgesetzt und später eigens überwacht sowie anhand der bisherigen Zielsetzungen und der Institutions- bzw. Unternehmenspolitik überprüft. Bei Bedarf werden sie korrigiert und aktualisiert. Die vorgenommenen Optimierungen werden zu neuen Standards und darüber hinaus Grundlage für den nächsten Zyklus. Der PDCA-Zyklus ermöglicht es so, einen neuen Prozess adäquat und effizient zu etablieren und durchzuführen. Durch risikobasiertes Denken bei der Planung eines Prozesses ermittelt die jeweilige Institution Faktoren, die zur Abweichung von den angestrebten Zielen führen könnten. Vorbeugende Kontrollmaßnahmen werden eingeführt, damit das Auftreten möglicher negativer Auswirkungen vermieden werden kann.

Die angestrebten kontinuierlichen Verbesserungen durch ein QM-System werden aufgrund ständiger Überprüfung schrittweise sichtbar. Beispiele hierfür sind verbesserte Feedbacks der Kunden, geringere Reklamations- und Beschwerderaten sowie bessere Ringversuchs- und Laborvergleichsergebnisse. Auch im Managementreview, bei dem Ergebnisse aus vorherigen Reviews mit dem aktuellen Zustand auf der Ebene der obersten Leitung einer Organisation verglichen werden, können Verbesserungen deutlich werden. In diesem häufig jährlich erstellten Review ist enthalten, ob die Qualitätsziele erreicht wurden, welche Trends in den Qualitätsindikatoren und -kennzahlen erkennbar sind und inwieweit interne und externe Auditergebnisse mögliche Abweichungen von Qualitätsvorgaben enthalten.

Auch wenn die Qualität in medizinischen Laboratorien gut gesichert ist, gibt es andere ebenso wichtige Bereiche in der Medizin, in denen es erst in den letzten Jahren zu einer Diskussion über einheitliche gemeinsame Standards kam. Seit der SARS-CoV-2-Pandemie und der damit verbundenen Steigerung des Qualitätsbewusstseins für die (fachärztlich‑)medizinische Labordiagnostik laufen sehr viele neue Qualitätsprojekte parallel sowie in Konkurrenz untereinander ab. Ein Beispiel hierfür ist die Zunahme an Projekten bezüglich mobiler Laboratorien, die der Krisenintervention und Kapazitätsverbesserung dienen sollen. Die Weltgesundheitsorganisation Region Europa (WHO/Europa) und das Global Outbreak Alert and Response Network (GOARN) arbeiten derzeitig an einheitlichen Standards, während gleichzeitig Kapazitäten aufgestockt werden und über Qualitätsmanagement in jedem Projekt diskutiert wird [19]. Ein weiteres Beispiel bezieht sich auf die Qualität in der medizinischen Forschung und wie diese garantiert werden kann. Hierfür gibt es von unterschiedlichen Institutionen, wie z. B. vom Berlin Institut of Health (BIH), bereits diverse Ansätze. Eine einheitliche Lösung für ein QM-System in der Forschung existiert aktuell nicht [20].

## Aufwand-Nutzen-Verhältnis von Maßnahmen in Qualitätssicherung und -management

Ein QM-System verursacht Kosten. Hier wird von intern anfallenden Kosten (v. a. Ressourcenbereitstellung) und externen Kosten (z. B. Zertifizierung, Akkreditierung) gesprochen [21]. In allgemeine Zahlen sind diese nicht fassbar, da sich Unternehmen u. a. in der Größe und im Umfang des Systems unterscheiden. Aus dem Betrachtungswinkel der medizinischen Labore führt die Etablierung und kontinuierliche Pflege eines Qualitätsmanagement- und Qualitätssicherungssystems mit nachfolgender Akkreditierung zu einer signifikanten Kostensteigerung, die sich derzeit nicht ausreichend in den Vergütungs- und Gebührenordnungen zur Refinanzierung der Laboratoriumsdiagnostik niederschlagen. Das ist bedeutsam, da diese Kosten weitestgehend durch die Umsetzung von Vorgaben aus Gesetzen und Verordnungen entstehen. Das gilt auch dann, wenn man auf die freiwillige Akkreditierung nach DIN EN ISO 15189 durch die DAkkS und so auf die damit verbundenen erheblichen Kosten der Verfahren zur Akkreditierung, Reakkreditierung und zwischenzeitlichen Überwachung verzichtet.

Nach einer groben Schätzung aus Perspektive des ALM e. V. (Verband der Akkreditierten Labore in der Medizin) entfallen nach Berechnungen ca. 10 % der Kosten in einem medizinischen Labor auf den Bereich von Qualitätsmanagement und Qualitätssicherung. Hierunter fällt auch ein Teil der Kosten, die durch die DAkkS im Rahmen des Akkreditierungsverfahrens verursacht werden. Der Nutzen von Maßnahmen zur Qualitätssicherung und zum Qualitätsmanagement liegt nach den Berichten und Erfahrungen aus Laboren ohne Zweifel in der Verbesserung der Prozess- und Ergebnisqualität laboratoriumsmedizinischer Untersuchungen und damit mittel- und unmittelbar in einer verbesserten Qualität der Patientenversorgung insgesamt. Insofern erscheint der Aufwand gerechtfertigt und im Sinne des Ziels sinnvoll und richtig.

Im Folgenden sind einige Aspekte aufgeführt, die höhere Ausgaben im Rahmen der Aufrechterhaltung eines QM-Systems verursachen:

- Ringversuche,
- konsequente Validierungen, Verifizierungen,
- Mess- und Prüfmittelüberwachungen,
- Personalkosten für die Etablierung, Wahrung und Weiterentwicklung des QM-Systems,
- erhöhter Schulungsbedarf der Mitarbeiter,
- ggf. anfallende Zertifizierungs- und Akkreditierungskosten.

Des Weiteren bemängeln Kritiker einen gesteigerten Zeitaufwand bei der Einführung sowie bei der Pflege eines solchen Systems. Zeit, Aufwand und Kosten, die für die Einführung eines QM-Systems in ein medizinisches Labor benötigt werden, zahlen sich bei einem funktionierenden System durch erfüllte Kundenanforderungen nach kompetenter Qualitätssicherung und durch Rechtssicherheit, beispielsweise durch die Probenrückverfolgbarkeit, aber wieder aus. Darüber hinaus kann ein QM-System die organisatorische Arbeit erleichtern und fördern. Im Folgenden sind einige Vorteile eines QM-Systems aufgelistet:

- größeres Vertrauen in die Qualität der Dienstleistungen seitens Kunden, Patienten und Öffentlichkeit,
- Rechtsicherheit bei der Nutzung von Inhousetests (s. Verordnung (EU) 2017/746 über In-vitro-Diagnostika (IVDR)),
- weniger Fehler und einfachere Fehlerbehebung,
- bessere Positionierung im Haftungsfall (Stand von Wissenschaft und Technik),
- deutliche Steigerung der Qualität der Labore und deren Prüfverfahren,
- höherer Wissenserhalt auch bei Personalwechsel und Abwesenheit,
- Erhöhung der Personalqualifikation,
- Geräte – richtig kalibriert und gewartet,
- geeignete und gültige Untersuchungsverfahren und -methoden,
- Verfahren zur Vorgehensweise der Datenerfassung und Berichterstattung.

Natürlich wird die tägliche Laborarbeit durch ein QM-System ebenso unterstützt, u. a. durch klare Verantwortlichkeiten (wie z. B. Autorisierungsplan) und aktuelle Betriebsanweisungen für Methoden und Geräte, in denen Verfahren klar festgelegt und niedergeschrieben sind.

## Zukünftige Herausforderungen für die Weiterentwicklung von Qualitätssicherung und -management

Eine zukünftige Herausforderung für die Weiterentwicklung von QS und QM wird die Schaffung von einheitlichen Standards in der allgemeinen Qualitätssicherung nicht nur in Deutschland, sondern EU-weit bzw. international sein. Während der SARS-CoV-2-Pandemie wurde die Frage gestellt, welche Länder eine Qualitätssicherung in der Diagnostik besitzen. Ein QM-System mit einer Akkreditierung nach der ISO 15189 oder ISO/IEC 17025 war hier von entschiedenem Vorteil. Bei Vorgaben, wie der Rili-BÄK, werden aus Sicht der Autoren zu wenig Überwachungskontrollen seitens der behördlichen Institutionen durchgeführt, um dies im Pandemiefall als alleinigen Standard zu verwenden. Zwar wurden im Verlauf der Pandemie u. a. das RKI sowie die akkreditierten Labore der ALM e. V. durch die zuständigen Überwachungsinstanzen zur Diagnostik von SARS-CoV‑2 mit positivem Kompetenznachweis überprüft. Dies erfolgte jedoch im Rahmen einer außerplanmäßigen Abfrage, da zu Beginn der SARS-CoV-2-Pandemie kaum CE-zertifizierte Reagenzien vorhanden waren. Das Ziel sollte sein, einen Überblick über die Tätigkeiten der Labore im Rahmen der Pandemie zu erhalten und u. a. festzustellen, ob diese unter den Überbegriff der Eigenherstellung fallen.

Die bisher durchgeführten Überwachungen seitens der zuständigen Behörden folgen einem bundesweit einheitlichen Konzept. Die Allgemeine Verwaltungsvorschrift zur Durchführung des Medizinproduktegesetzes [22] regelt u. a. klar die übergeordneten Grundsätze der Überwachung, die Durchführung von Inspektionen, das Verfahren bei festgestellten Mängeln und die Etablierung eines Systems zur Qualitätssicherung. Hier ist die Zentralstelle der Länder für Gesundheitsschutz bei Arzneimitteln und Medizinprodukten (ZLG) als Koordinierungsstelle der Länder u. a. für die Weiterentwicklung des Qualitätssicherungssystems in der Medizinprodukteüberwachung zuständig. Es wurden 6 Fachexpertengruppen (FEG) einberufen: 1) Qualitätssicherung, 2) Inverkehrbringen, 3) klinische Prüfungen, 4) Betreiben und Anwenden, 5) hygienische Aufbereitung und 6) medizinische Laboratorien. Zu den Aufgaben der FEG zählen u. a. die Erarbeitung eines Qualitätssicherungshandbuchs zur Medizinprodukteüberwachung und von Verfahrensanweisungen sowie die Anpassung der Checklisten an eine geänderte Rechtslage. Sie klären Vollzugsfragen, um einen bundesweit einheitlichen Vollzug zu gewährleisten. Alle Qualitätssicherungsdokumente werden von der AGMP (Arbeitsgruppe Medizinprodukte: Gremium seit 2002, Koordination der Zusammenarbeit der Länder) auf ministerialer Ebene beschlossen, von den Länderministerien zur Anwendung freigegeben und von den Vollzugsbehörden in Kraft gesetzt. Das Qualitätssicherungshandbuch für die Medizinprodukteüberwachung soll sicherstellen, dass der Vollzug durch die Länder angemessen und einheitlich erfolgt. Es ist ein Überwachungsplan anzufertigen und jährlich von den Vollzugsbehörden über die ZLG an die AGMP Bericht zu erstatten. Die Überwachungspläne richten sich nach dem „Konzept zur Umsetzung der den Obersten Landesbehörden durch die MPGVwV zugeschriebenen Aufgaben“, einem behördeninternen Dokument. Dieses Konzept ist risikobasiert. Die Einstufung der medizinischen Labore erfolgt in die Kategorie mit dem niedrigsten Risiko. Zum Vergleich erhielten die höchste Risikostufe z. B. die Labore der Krankenhäuser. Weitere als risikoreich eingestufte Bereiche sind z. B. die Sofortdiagnostik und die Schnelltestzentren für SARS-CoV‑2, in welchen Untersuchungen nicht von Fachleuten und z. T. in nicht klimatisierten Zelten durchgeführt werden. Sollen folglich vermehrte Kontrollen bzgl. der Rili-BÄK als einheitlichen Standard durchgeführt werden, ähnlich zu den Überwachungen der Akkreditierungen, müsste die Risikoeinstufung der medizinischen Labore angepasst werden.

Die oben genannten Normen sowie das System der Akkreditierung sind bewährt und beinhalten einen von unabhängigen Dritten bestätigten und international anerkannten Nachweis der Kompetenz. Das RKI beteiligt sich u. a. auch an inter- und nationaler Gremienarbeit (wie den Revisionen ISO/IEC 17025, ISO/IEC 17043 und ISO 15189) mit dem Ziel, die Akkreditierungsinfrastruktur auch weltweit weiterzuentwickeln. Derzeit werden die Normen ISO 15189 und ISO/IEC 17043 u. a. auf internationaler Ebene einer Revision unterzogen. Sie dienen einer Harmonisierung der Struktur- und Managementsystemanforderungen für Anwender, die medizinische sowie Prüf- und Kalibrierlaboratorien betreiben und/oder Referenzmaterialien herstellen.

Aus Sicht der medizinischen Laboratorien ist grundsätzlich eine „Überregulierung“ durch Qualitätsmanagement- und Qualitätssicherungsrichtlinien zu vermeiden. Die Weiterentwicklung der internationalen Normen, allen voran der für die medizinischen Laboratorien relevanten ISO 15189 sowie ISO/IEC 17025, sollte stets die mit den Normen verfolgten Ziele im Blick behalten und die Aspekte regeln, die einen signifikanten Einfluss auf die mit den Normen verbundenen Ziele haben. Das gilt auch für die nationale Umsetzung der Normen, die maßgeblich von deutschen Spezifika (Gesetzgebung, Gremien, Verbänden) beeinflusst wird. Hierfür ist zukünftig neben einer bereits existierenden Mitarbeit des RKI, der Technischen Hochschule Lübeck und der DAkkS eine verstärkte Beteiligung von Vertretern der medizinischen Laboratorien aus Deutschland in den Normierungsgremien zu empfehlen.

In den vergangenen Jahren lässt sich eine gewisse Fehlentwicklung beobachten, die in erster Linie Aufwand und damit auch Kosten zur Aufrechterhaltung der Akkreditierung berührt und weniger zur Verbesserung der Qualität beiträgt. Das betrifft im Konkreteren die zunehmende Bürokratie bei den Akkreditierungsverfahren. Das Grundprinzip der freiwilligen Akkreditierung hat sich in Deutschland bewährt und sollte beibehalten werden. Damit verbunden ist aber das Risiko, dass medizinische Labore unter Aufrechterhaltung und eigenständiger Fortentwicklung der eigenen QM-/QS-Systeme auf den formalen Prozess der DAkkS-Akkreditierung und damit auf den Nachweis ihrer Kompetenz durch eine unabhängige international anerkannte Institution verzichten.

Ein alternativ genutztes Konzept zur anerkannten Qualitätssicherung ist das European-Foundation-for-Quality-Management-Modell (EFQM-Modell; [23]). Es ist ein Managementmodell, welches Organisationen bei den Verbesserungen ihrer Leistungen unterstützen soll. Nach dem Modell ist das Erreichen der Zufriedenheit aller Interessengruppen (Kunden, Beschäftigte, Lieferanten, Partner) in den Mittelpunkt des Handelns zu stellen. Es dient als Diagnoseinstrument für die Organisation, um die eigenen Stärken, Schwächen und Verbesserungspotenziale zu erkennen und die Strategie darauf auszurichten [24].

## Fazit

Ein umfassendes QM-System unterstützt die Arbeit der in einem Institut oder (fach-)ärztlich-medizinischen Labor Tätigen maßgeblich. Es hat viele Vorteile, wie klare Verantwortungsbereiche, genau festgelegte Prozesse und einen präzisen nachverfolgbaren Weg für die Handhabung von klinischen Proben einschließlich der Aspekte der kontinuierlichen Verbesserung. Die Wahrung des Vertrauens in die Leistungsfähigkeit von medizinischen Laboratorien und auch in die sie tragenden Institute ist für eine bestmögliche medizinische Versorgung der Bevölkerung von großer Bedeutung. Insbesondere nationale und internationale Fachempfehlungen (u. a. [25, 26]) gelten als wesentliche Leitlinien in der Pandemie für die Bevölkerung, für die Ärzteschaft mit den (fach-)ärztlichen medizinischen Laboratorien, für den Öffentlichen Gesundheitsdienst und auch für die politisch Verantwortlichen auf Landes- und Bundesebene. Im Sinne der Patientensicherheit und des Vertrauens in Sicherheit und Qualität der Diagnostik sollten in Deutschland einheitliche hohe Standards verpflichtend sein. Dies erfordert keineswegs die in anderen Ländern bereits etablierte Verpflichtung zur Akkreditierung. Vielmehr kommt es auf die Etablierung eines konsentierten Qualitätsstandards an, auf deren Einhaltung sich alle fokussieren. Nach der MPBetreibV hat derjenige, der laboratoriumsmedizinische Untersuchungen durchführt, ein Qualitätssicherungssystem nach dem Stand der medizinischen Wissenschaft und Technik einzuführen. Dabei stellt die Beachtung der Rili-BÄK eine wichtige Grundlage dar. Zwischen den dort aufgeführten grundlegenden Anforderungen an die Qualitätssicherung laboratoriumsmedizinischer Untersuchungen und den Inhalten der DIN EN ISO 15189 gibt es vielfältige inhaltliche Übereinstimmungen. In der MPBetreibV wird auf die Rili-BÄK vom 19.09.2014 Bezug genommen. Dies hat den Hintergrund, dass eine zweijährige Übergangszeit in Abschnitt F der Rili-BÄK von Oktober 2019 festgelegt ist. Im Jahr 2022 ist geplant, die MPBetreibV diesbezüglich anzupassen.

Ein Akkreditierungsprozess ist zwar prinzipiell für alle medizinischen Laboratorien anwendbar, gleichwohl nicht immer leicht umsetzbar. Darüber hinaus wurde bereits auf die steigenden Kosten und die erhöhte Bürokratie in den letzten Jahren hingewiesen. Auch aus der Perspektive fachärztlicher medizinischer Labore, die bereits akkreditiert sind, ergibt sich die Notwendigkeit, das Kernziel der Akkreditierung im Blick zu behalten und Inhalte sowie das Vorgehen des Akkreditierungsverfahrens darauf abzustellen. Eine verbesserte Vorababwägung zwischen Nutzen und Aufwand zur Lösungsfindung ist hier wünschenswert, um den tatsächlichen Mehrwert einer Akkreditierung zu erhalten.

Weiterhin wurde im Jahr 2020 die Bedeutung der Qualitätssicherung in Laboratorien und damit auch die Akkreditierung national und international auf ein höheres Niveau gehoben. Gerade jetzt in der Pandemiesituation sind Befunde von akkreditierten Laboratorien von essenzieller Bedeutung, auch im Rahmen der allgemeinen Maßnahmen, so z. B. für die internationalen Reisebestimmungen. Auch in diesem Zusammenhang diente das DAkkS-ILAC-Symbol weltweit als Erkennungszeichen und wird zur Visualisierung der einheitlichen Standards verwendet. Zum Beispiel tragen die medizinischen Laboratorien der ALM e. V. und das RKI das DAkkS/ILAC-Symbol der Akkreditierung nach DIN EN ISO 15189 und zum Teil nach DIN EN ISO/IEC 17025 [27]. Eine Akkreditierung durch eine unabhängige Stelle belegt die Kompetenz und Normenkonformität, die Vertrauen in die Sicherheit und Qualität der Patientenversorgung auf internationalem Niveau gewährleistet.

### Infobox 1 Akkreditierung

Eine Akkreditierung ist die „Bestätigung durch eine nationale Akkreditierungsstelle, dass eine *Konformitätsbewertungsstelle* die in harmonisierten Normen festgelegten Anforderungen und gegebenenfalls zusätzliche Anforderungen … erfüllt, um eine spezielle Konformitätsbewertungstätigkeit durchzuführen“ (Definition laut Verordnung (EG) Nr. 765/2008).

- In Deutschland werden Akkreditierungen von der Deutschen Akkreditierungsstelle (DAkkS) durchgeführt.
- Akkreditierte Laboratorien werden regelmäßig einer Überwachungs- und Wiederholungsbegutachtung unterzogen, um die ständige Einhaltung von Anforderungen sicherzustellen und um zu prüfen, ob die Standards ihrer fachlichen Kompetenz aufrechterhalten bleiben.

## References

[1] DIN EN ISO 15189:2014-11 (2014). Medizinische Laboratorien – Anforderungen an die Qualität und Kompetenz.

[2] DIN EN ISO/IEC 17025:2018-03 (2018). Allgemeine Anforderungen an die Kompetenz von Prüf- und Kalibrierlaboratorien.

[3] Medizinprodukte-Betreiberverordnung in der Fassung der Bekanntmachung vom 21. August 2002 (BGBl. I S. 3396), die zuletzt durch Artikel 7 der Verordnung vom 21. April 2021 (BGBl. I S. 833) geändert worden ist

[4] Landesamt für Mess- und Eichwesen Berlin Brandenburg http://lme.berlin-brandenburg.de/sixcms/detail.php/876673. Zugegriffen: 7. Okt. 2021

[5] Regierungspräsidium Tübingen, Abteilung 10 Eich- und Beschusswesen https://rp.baden-wuerttemberg.de/rpt/abteilungen/abteilung-10/. Zugegriffen: 7. Okt. 2021

[6] Regierungspräsidium Darmstadt, Fachzentrum MPG im RP Kassel https://rp-darmstadt.hessen.de/sicherheit/produktsicherheit/medizinprodukte/fachzentrum-mpg-im-rp-kassel. Zugegriffen: 7. Okt. 2021

[7] Bezirksregierung Köln https://www.bezreg-koeln.nrw.de/brk_internet/leistungen/abteilung02/24/medizinprodukte/anwendung/index.html. Zugegriffen: 7. Okt. 2021

[8] Sozialgesetzbuch (SGB) Fünftes Buch (V) – Gesetzliche Krankenversicherung – (Artikel 1 des Gesetzes v. 20. Dezember 1988, BGBl. I S. 2477); zuletzt geändert durch Art. 2a G v. 28. Mai 2021 I 1174

[9] MTA-Gesetz vom 2. August 1993 (BGBl. I S. 1402), das zuletzt durch Artikel 34 des Gesetzes vom 15. August 2019 (BGBl. I S. 1307) geändert worden ist

[10] Arzneimittelgesetz in der Fassung der Bekanntmachung vom 12. Dezember 2005 (BGBl. I S. 3394), das zuletzt durch Artikel 10 des Gesetzes vom 10. August 2021 (BGBl. I S. 3436) geändert worden ist

[11] Bundesärztekammer (2021) (Muster‑)Berufsordnung für die in Deutschland tätigen Ärztinnen und Ärzte. https://www.bundesaerztekammer.de/fileadmin/user_upload/downloads/pdf-Ordner/Recht/_Bek_BAEK_MBO-AE_Online_final.pdf. Zugegriffen: 7. Okt. 2021

[12] Gemeinsamer Bundesausschuss (2020) Richtlinie des Gemeinsamen Bundesausschusses über grundsätzliche Anforderungen an ein einrichtungsinternes Qualitätsmanagement. https://www.g-ba.de/downloads/62-492-2309/QM-RL_2020-09-17_iK-2020-12-09.pdf. Zugegriffen: 7. Okt. 2021 (für Vertragsärztinnen und Vertragsärzte, Vertragspsychotherapeutinnen und Vertragspsychotherapeuten, medizinische Versorgungszentren, Vertragszahnärztinnen und Vertragszahnärzte sowie zugelassene Krankenhäuser)

[13] Bundesärztekammer (2019). Richtlinie der Bundesärztekammer zur Qualitätssicherung laboratoriumsmedizinischer Untersuchungen. Dtsch Arztebl.

[14] TRBA 100 Schutzmaßnahmen für Tätigkeiten mit biologischen Arbeitsstoffen in Laboratorien. Ausgabe Oktober 2013 GMBl 2014, Nr. 51/52 vom 17.10.2013, letzte Änderung vom 02.05.2018, GMBl Nr. 15

[15] TRBA 250 Biologische Arbeitsstoffe im Gesundheitswesen und in der Wohlfahrtspflege. Ausgabe März 2014 GMBl 2014, Nr. 10/11 vom 27.03.2014, letzte Änderung vom 02.05.2018, GMBl Nr. 15

[16] Care Quality Commission (2020) Joint statement on the move to UKAS accreditation and removal of the CQC registration requirement for providers of commercial coronavirus testing. 10.1007/978-3-319-76403-0. Zugegriffen: 27. Aug. 2021

[17] Kassenärztliche Bundesvereinigung (2021) Qualitätsbericht 2020. https://www.kbv.de/media/sp/KBV_Qualitaetsbericht_2020.pdf. Zugegriffen: 20. Aug. 2021

[18] Akkreditierungssymbolverordnung vom 15. Dezember 2009 (BGBl. I S. 3870)″

[19] WHO Europe (2021) Guidance for rapid response mobile laboratory (RRML) classification. https://www.euro.who.int/en/health-topics/health-emergencies/publications/2021/guidance-for-rapid-response-mobile-laboratory-rrml-classification-2021. Zugegriffen: 7. Okt. 2021

[20] Dirnagl U (2018). Quality management for academic laboratories: burden or boon? Professional quality management could be very beneficial for academic research but needs to overcome specific caveats. EMBO Rep.

[21] Tittel K, Weber U, Durian G (2021) Einführung eines Qualitätsmanagementsystems. IHK Schwaben. Nr. 75048. https://www.schwaben.ihk.de/blueprint/servlet/resource/blob/555250/4b6dcde1255d23afe976993d39a6e78a/merkblatt-qualitaetsmanagementsystem-data.pdf. Zugegriffen: 23. Nov. 2021

[22] Allgemeine Verwaltungsvorschrift zur Durchführung des Medizinproduktegesetzes (Medizinprodukte-Durchführungsvorschrift – MPGVwV) vom 18. Mai 2012

[23] Bundesministerium des Innern Organisationshandbuch. https://www.orghandbuch.de/OHB/DE/Organisationshandbuch/7_Management/79_Qualitaetsmanagement/792_EFQM-Modell/efqm-modell-node.html. Zugegriffen: 7. Okt. 2021

[24] Otte K, Gottschall K (2016) Kennen Sie EFQM? Kontinuierliche Verbesserung der Qualität als Ziel. Deutscher Ärzteverlag, MTA Dialog. http://www.inquam.de/fileadmin/user_upload/MTA_07_2016-Heft_17_Otte_Gottschall.pdf. Zugegriffen: 23. Nov. 2021

[25] Robert Koch-Institut (2020) Bericht zur Optimierung der Laborkapazitäten zum direkten und indirekten Nachweis von SARS-CoV‑2 im Rahmen der Steuerung von Maßnahmen. https://www.rki.de/DE/Content/InfAZ/N/Neuartiges_Coronavirus/Laborkapazitaeten.html. Zugegriffen: 7. Okt. 2020

[26] WHO Coronavirus disease (COVID-19) pandemic. https://www.who.int/emergencies/diseases/novel-coronavirus-2019. Zugegriffen: 7. Okt. 2021

[27] Brünschwitz S, Kleymann-Hilmes J, Mielke M, Schaade L, Wieler L (2020). The quality management system of the Robert Koch Institute. 17th IMEKO TC 10 and EUROLAB Virtual Conference “Global Trends in Testing, Diagnostics & Inspection for 2030”.

